# Cued Reactivation of Motor Learning during Sleep Leads to Overnight Changes in Functional Brain Activity and Connectivity

**DOI:** 10.1371/journal.pbio.1002451

**Published:** 2016-05-03

**Authors:** James N. Cousins, Wael El-Deredy, Laura M. Parkes, Nora Hennies, Penelope A. Lewis

**Affiliations:** 1 School of Psychological Sciences, University of Manchester, Manchester, United Kingdom; 2 Cognitive Neuroscience Laboratory, Duke-NUS Graduate Medical School, Singapore; 3 School of Biomedical Engineering, University of Valparaiso, Valparaiso, Chile; 4 Centre for Imaging Sciences, University of Manchester, Manchester, United Kingdom; 5 Department of Systems Neuroscience, University Medical Center Hamburg-Eppendorf Martinistr, Hamburg, Germany; Radboud Universiteit Nijmegen, NETHERLANDS

## Abstract

Sleep plays a role in memory consolidation. This is demonstrated by improved performance and neural plasticity underlying that improvement after sleep. Targeted memory reactivation (TMR) allows the manipulation of sleep-dependent consolidation through intentionally biasing the replay of specific memories in sleep, but the underlying neural basis of these altered memories remains unclear. We use functional magnetic resonance imaging (fMRI) to show a change in the neural representation of a motor memory after targeted reactivation in slow-wave sleep (SWS). Participants learned two serial reaction time task (SRTT) sequences associated with different auditory tones (high or low pitch). During subsequent SWS, one sequence was reactivated by replaying the associated tones. Participants were retested on both sequences the following day during fMRI. As predicted, they showed faster reaction times for the cued sequence after targeted memory reactivation. Furthermore, increased activity in bilateral caudate nucleus and hippocampus for the cued relative to uncued sequence was associated with time in SWS, while increased cerebellar and cortical motor activity was related to time in rapid eye movement (REM) sleep. Functional connectivity between the caudate nucleus and hippocampus was also increased after targeted memory reactivation. These findings suggest that the offline performance gains associated with memory reactivation are supported by altered functional activity in key cognitive and motor networks, and that this consolidation is differentially mediated by both REM sleep and SWS.

## Abstract 2

Slow-wave sleep and rapid eye movement sleep are associated with the reactivation and consolidation of a motor skill within distinct brain networks.

## Author Summary

After a motor skill is learned, the memory undergoes "offline" processing so that improvement occurs even without further practice. Sleep has been shown to enhance this consolidation and, in the process, to reorganize the brain regions involved. However, it remains unclear how sleep does this, and whether different sleep stages have different contributions. One popular idea is that the memory trace is reactivated during slow-wave sleep—a period of sleep characterized by synchronized activity at a slow frequency and high amplitude, as recorded by electroencephalography (EEG)—which drives memory reorganization within the brain. To test this in humans, we took advantage of "targeted memory reactivation," where replay of specific memories is cued by presentation of a sound that was present during learning. After sleep, motor performance was faster for cued memories, suggesting that the trace was consolidated during sleep. Coupled with this, brain activation and connectivity in several motor-learning areas was enhanced for the cued memory. Furthermore, some changes in brain activity were associated with time spent in slow-wave sleep, while others were associated with time spent in rapid-eye movement sleep. These observations provide further insight into sleep's unique role in memory consolidation by showing that offline skill enhancement depends on the reactivation of specific memories, and the associated changes in neural activity may rely upon processing that unfolds across different stages of sleep.

## Introduction

Memory consolidation begins the moment new information is encoded and is a process where initially fragile memories are stabilised, strengthened, and reorganised in the brain [1]. Learning a new motor skill, for example, requires episodes of repeated practice, and is also supported by offline consolidation periods where stabilisation and gains in performance are observed [2]. Such performance improvement is reflected by plastic changes within key motor memory networks over time [3–5], and several studies contrasting sleep and wake consolidation periods suggest that sleep provides the optimal conditions for this offline processing to occur [6–13].

The spontaneous reactivation of cerebral activity after learning is hypothesised to underscore such plasticity during sleep and the associated performance gains [14–17]. This “memory replay” has been observed in multiple brain regions during sleep in rodents [18–23] and humans [24–26]. Moreover, neural replay has been linked to sleep-dependent improvements in skilled motor movements [21], while indirect disruption of this replay impacts upon spatial learning [22] and synaptic plasticity [23]. Targeted memory reactivation (TMR) during sleep, where the replay of specific memories can be cued via presentation of learning related sounds or odours [19,27–34], provides further behavioural evidence that reactivation supports the consolidation of procedural skill in humans [28,30,31]. However, it is unknown whether these performance improvements after TMR are supported by underlying changes in activity within motor memory networks, changes that provide an indirect measure of underlying plasticity. The neurophysiological correlates of consolidation after TMR have been demonstrated for declarative memories [32,33], but not procedural, and it remains unclear how they relate to the behavioural effects of TMR.

Overnight procedural memory consolidation is linked to enhanced functional activation within striatum, hippocampus, cerebellum, and motor cortical regions, as well as striato-hippocampal and medial prefrontal-hippocampal (mPFC-HPC) connectivity [7–12,35]. Interactions between these networks are thought to assist the development of a refined motor representation and subsequently guide sleep-dependent consolidation [35]. Given that reactivation is thought to drive the plasticity associated with sleep [14–17], we hypothesised that TMR would modulate such changes in functional activity and connectivity.

The role of rapid eye movement (REM) sleep in procedural memory reactivation and consolidation is debated [36]. REM was initially linked with consolidation of procedural memory tasks [37–39], and learning-related brain regions have been shown to reactivate during REM [24,25]. However, several studies now suggest REM may not be critical for procedural memory [36,40,41]. Furthermore, recent TMR studies link reactivation during slow-wave sleep (SWS) [28,30] and non-rapid eye movement (NREM) [31] with consolidation of motor tasks, while reactivation work in rodents focuses largely on NREM [18–21,23,42]. The interleaved cycles of REM and NREM periods could be important for consolidation [14,43], with REM providing synaptic consolidation of memories that were already reactivated during NREM [14], yet data linking REM with memory reactivation is lacking. In this paper, we set out to explore the role of both REM and NREM in consolidation by examining how individual differences in REM and SWS influence brain function after TMR.

**Fig 1:**
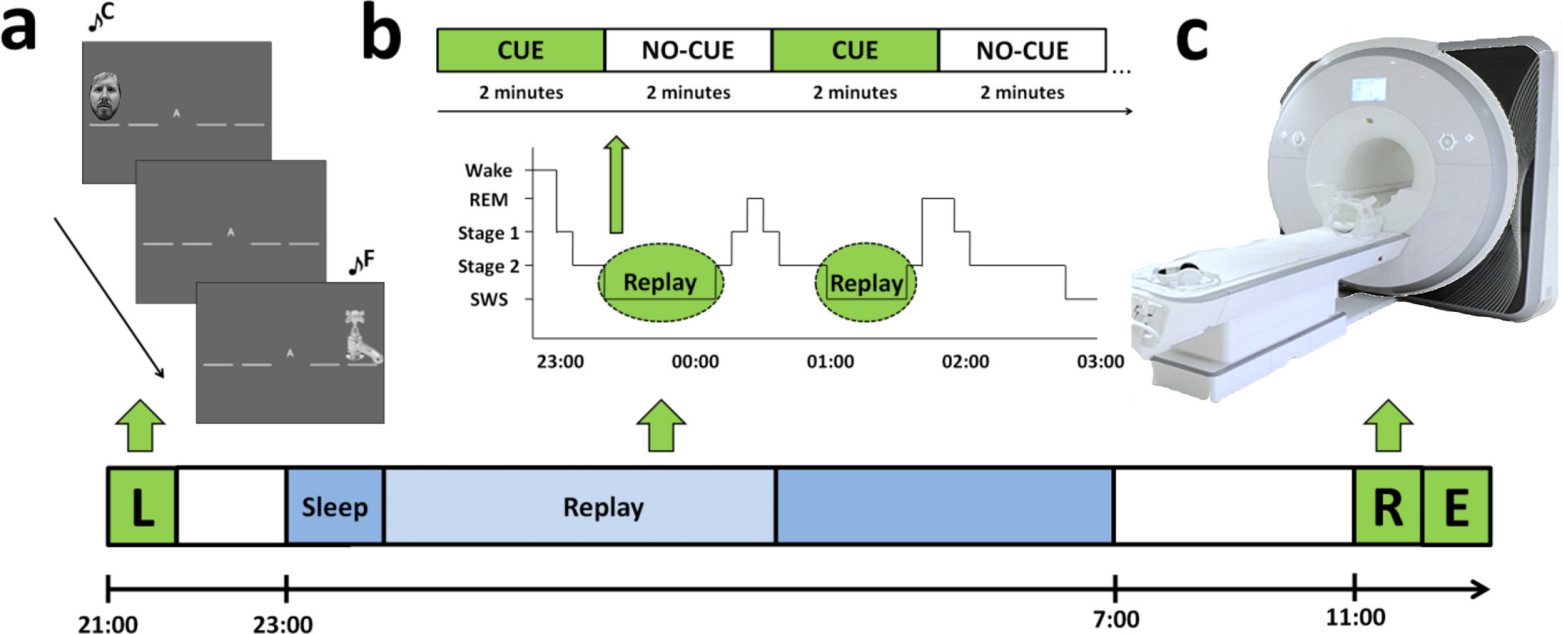
Schematic of experiment design. (a) Learning (L) of the SRTT task consisted of interleaved blocks of the cued and uncued sequence, and also random blocks. (b) The cued sequence is replayed during periods of SWS in groups of 12 sequences (CUE) and equivalent periods of silence (NO-CUE). (c) Retest (R) of the SRTT takes place the following morning in the MRI scanner, followed shortly afterwards by the explicit memory test outside of the scanner.

We aimed to test this by cueing a modified version of the serial reaction time task (SRTT) [44] during SWS and measuring subsequent differences in functional activity and connectivity during retest the following day using functional magnetic resonance imaging (fMRI). Participants were required to react to visual stimuli appearing at one of four possible positions on screen, corresponding to four buttons on a response pad. Stimuli followed two repeating 12-item patterns presented in separate blocks. The two sequences were associated with different sets of tones (either high or low in pitch, Fig 1). One sequence (cued) was reactivated during nocturnal SWS by replaying the associated tones in sequence, while the other sequence (uncued) was not. Brain activity and behavioural measures of speed and accuracy were compared while performing cued and uncued sequences at postsleep retest. We were particularly interested in exploring how these differences in functional activity were influenced by SWS and REM. We predicted: (1) TMR would lead to faster reaction times (RTs) for the cued sequence. (2) TMR would result in a cueing-dependent increase in cerebral activation within structures that are important for sequence consolidation, namely the striatum, hippocampus, cerebellum, medial prefrontal cortex (mPFC), and motor cortical areas [7–12,35]. (3) Since SWS duration is associated with consolidation [45] and TMR effects [46,47], we predicted that changes in activation within the aforementioned structures would be associated with SWS. (4) Lastly, because procedural memory consolidation has also been linked with REM sleep [37–39] and stage 2 sleep [48,49], while the neurophysiological correlates of consolidation have also been linked with overnight changes in performance [7,8,10–12,35,50], we expected that functional activation changes might also be related to these factors.

## Results

### Behavioural Analysis

#### RTs

Firstly, we confirmed that sequence learning occurred prior to sleep by showing that RTs were significantly faster for sequence trials compared to random trials for both cued, t(21) = 9.2, *p* < 0.001, and uncued, t(21) = 9.22, *p* <0.001, sequences (S1 Text, S1 Data). Table 1 provides mean RT and accuracy for all analyses. Crucially, we also demonstrated that prior to sleep there was no significant difference between RTs for cued and uncued sequences, t(21) = 1.05, *p* = 0.31, or cued and uncued random trials, t(21) = 0.22, *p* = 0.83; therefore, postsleep differences are attributable to TMR of the cued sequence.

**Table 1:** RT and accuracy scores for cued and uncued sequence performance.

		RT (ms)		Errors (%)	
		Cued	Uncued	Cued	Uncued
**SRTT Test Blocks (Mean ± Standard Deviation)**					
***Presleep***					
**Learning**	**Sequence**	357.2 ± 51.0	351.3 ± 47.1	7.8 ± 4.7	5.8 ± 3.3
	**Random**	421.4 ± 31.6	422.4 ± 34.1	6.9 ± 4.0	7.6 ± 3.5
	**Difference**	64.2 ± 32.7	71.1 ± 36.2	0.9 ± 6.2	1.8 ± 5.3
***Postsleep***					
**Early**	**Sequence**	335.8 ± 39.9	342.7 ± 44.9	3.8 ± 2.5	4.7 ± 3.3
**Late**	**Sequence**	307.2 ± 51.7	304.6 ± 54.1	5.3 ± 2.8	4.7 ± 3.2
	**Random**	392.2 ± 30.2	400.0 ± 32.8	6.2 ± 3.6	6.7 ± 4.7
	**Difference**	84.9 ± 40.7	95.4 ± 46.1	0.9 ± 3.1	2.0 ± 3.4
***Improvement***					
**Early**	**Sequence**	21.4 ± 24.0	8.6 ± 23.8	3.9 ± 4.0	1.6 ± 3.1
**Late**	**Sequence**	50.0 ± 25.6	46.8 ± 24.5	2.4 ± 3.0	1.1 ± 2.2
	**Random**	29.3 ± 22.1	22.5 ± 24.3	0.7 ± 5.1	0.9 ± 5.7
	**Difference**	20.7 ± 32.8	24.3 ± 33.5	1.8 ± 6.1	0.2 ± 6.6

Early: mean performance during the first four blocks of each sequence at retest.

Late: mean performance during the last four blocks of each sequence at retest.

Improvement: the difference between presleep and postsleep performance.

**Fig 2:**
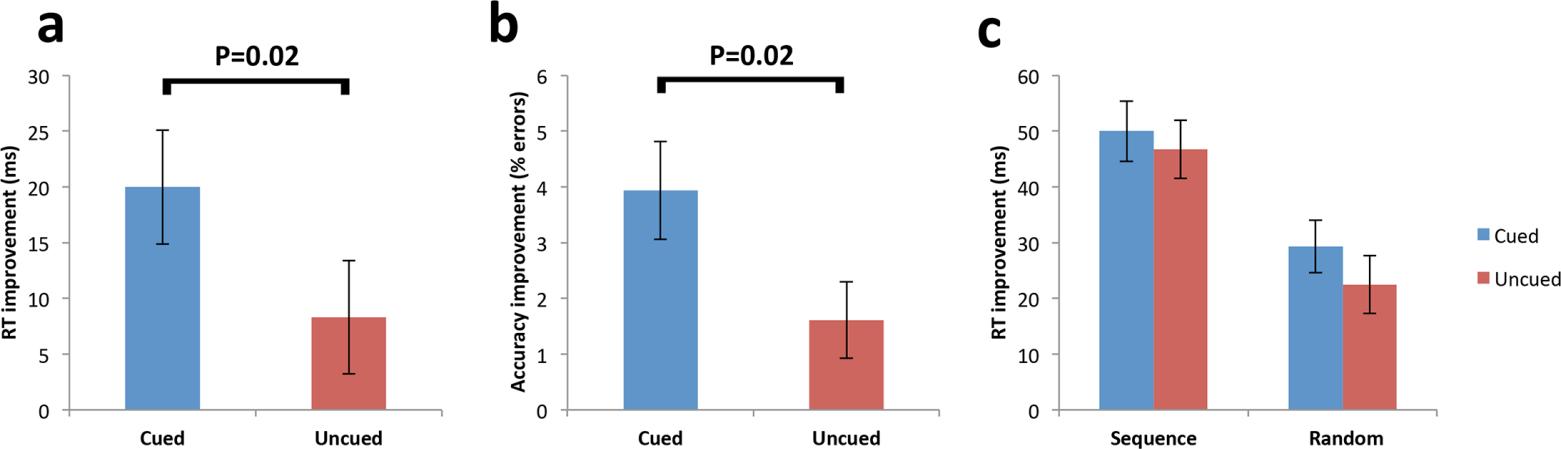
Performance improvement at retest. **(a)** Comparison of presleep sequence performance to early blocks of sequence retest showed a significant cueing effect. **(b)** Accuracy improvement was also significantly greater for the cued sequence at early blocks. **(c)** Performance improvement for both sequences was comparable at late sequence blocks and random blocks that followed. Error bars represent standard error of the mean (SEM) (S1 Data).

Next, we explored early improvement in RTs due to TMR effects by subtracting initial blocks of sequence retest from the final blocks of sequence learning (presleep performance), providing a measure of “early sequence improvement.” Here, we found improvement for the cued sequence was significantly greater than the uncued, t(21) = 2.46, *p* = 0.02 (Fig 2A). Cued sequence improvement was found to be significantly different from 0, t(21) = 4.18, *p* < 0.001, while uncued sequence improvement was not, t(21) = 1.7, *p* = 0.1.

Next, we compared cued and uncued sequence performance during the last blocks of the retest (late sequence improvement) and found that the advantage for the cued sequence was no longer present, t(21) = 0.58, *p* = 0.57. We also took performance on these late sequence blocks and subtracted it from performance on random blocks that followed immediately after them at retest. This provides a measure of sequence skill alone by accounting for any performance improvement associated with learning the mapping between stimuli and button responses. The cued and uncued sequences did not significantly differ for this “sequence-specific improvement” measure, t(21) = −0.45, *p* = 0.66.

To summarise, we found a behavioural cueing effect at the beginning of retest, but cued and uncued sequence performance equalised across the duration of the retest block such that this was no longer present in late sequence blocks. In light of our previous findings [28] and other studies [31,51–54], we expect this early improvement reflects an improvement in sequence learning rather than visuomotor mapping.

#### Error rates

Accuracy is typically high for motor sequence learning (MSL) tasks and does not change substantially across practice or offline [31,51–54], and the same was true across our experiment (4.7%–7.8% trials). Performance of the SRTT can be seen as a trade-off between accuracy and speed; therefore, we analysed errors across the same test blocks as the RT analysis to ensure our cueing effects for RT were not due to an alteration in the speed–accuracy trade-off (Table 1). One participant was excluded due to corrupted error data, leaving *n* = 21.

There was a trend for more errors to be made for the cued sequence relative to the uncued sequence prior to sleep, t(20) = 1.86, *p* = 0.08, although this was a difference of only 2% between cued and uncued errors (Table 1). Random trials showed no difference between cued and uncued prior to sleep, t(20) = 0.9, *p* = 0.38.

After sleep, there was a significantly greater error rate improvement for the cued relative to the uncued sequence at early blocks, t(20) = 2.46, *p* = 0.02. Due to the trend for more cued sequence errors prior to sleep, we also compared absolute error rates after sleep irrespective of presleep performance, and these did not significantly differ, t(20) = −1.17, *p* = 0.26. Taken together, we cannot conclude that cueing influenced accuracy as well as RT, since the cued sequence arguably had more room to improve. Importantly, however, this shows that the significant RT enhancement for the cued sequence across early blocks represents a gain in speed, rather than being the result of a shift in the speed–accuracy trade-off, because errors were actually less for the cued (M = 3.8 ± 2.5) than the uncued sequence (M = 4.7 ± 3.3).

#### Replay & sleep parameters

Considering all sleep electroencephalography (EEG) as 30 s epochs, 96% of CUE periods and 97% of NO-CUE periods were in SWS. All others were stage 2 and excluded from further EEG analyses. Duration of sleep stages and total sleep are displayed in Table 2. The number of sequences replayed varied across participants (Mean = 153.4 ± 49.4). Combining CUE/NO-CUE periods gave 51.1 ± 16.5 min mean replay time. Cues were played opportunistically during SWS periods in the first half of the night, and this was reflected in a positive correlation between the number of replayed sequences and SWS duration (r = 0.49, *p* = 0.03). Of note, no behavioural performance measures correlated significantly with the number of replays or time spent in sleep stages (*p* > 0.05). Comparison of arousal events during replay (CUE) and after replay (NO CUE) showed no evidence that sounds disrupted sleep: arousals (*p* = 0.4), movements (*p* = 0.6), or awakenings (*p* = 1). Mean alpha power at occipital electrodes can indicate arousal, and we found this did not differ between CUE/NO-CUE periods, t(18) = 0.97, *p* = 0.35, suggesting sounds did not disrupt sleep. Lastly, no participants reported hearing tones during the night.

**Table 2:** Total time spent in sleep stages.

	Duration (min ± Standard Deviation)
**Stage 1**	32.7 ± 26.7
**Stage 2**	217.9 ± 46
**SWS**	102.1 ± 34.3
**REM sleep**	84.2 ± 27.3
**Total Sleep Time**	440 ± 65

### Functional Imaging Analysis

**Fig 3:**
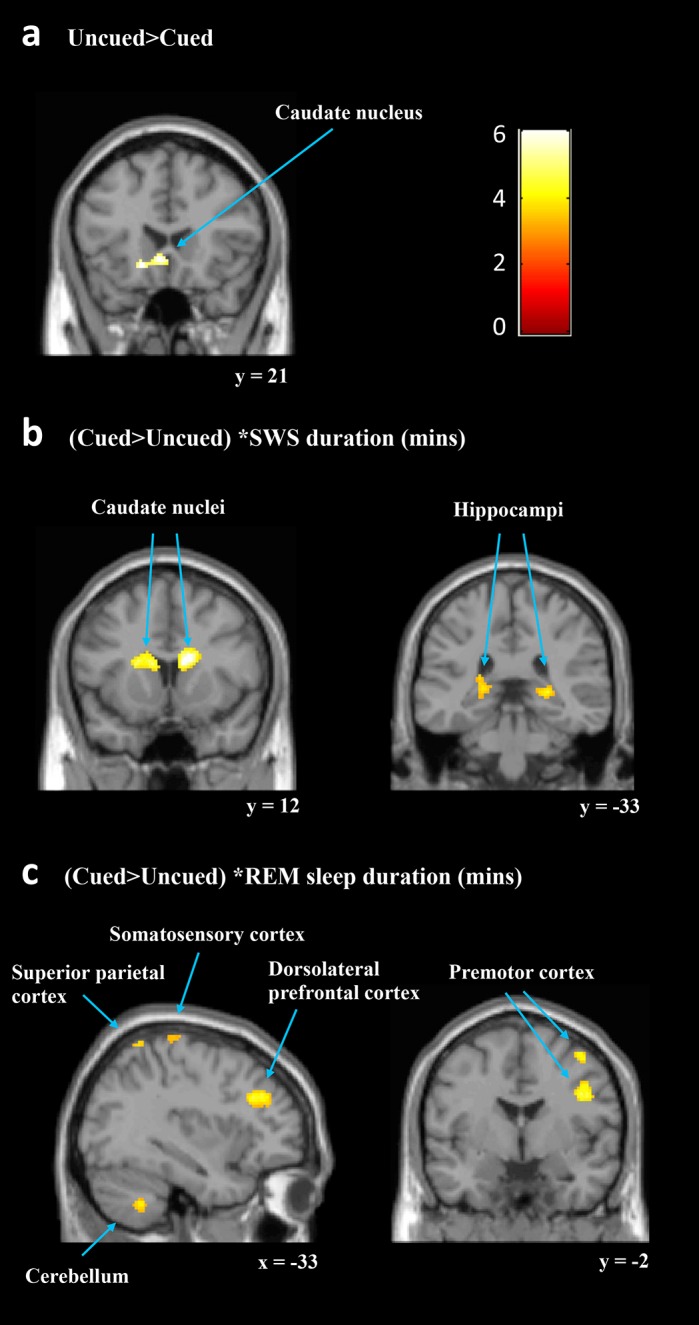
Changes in brain activity after targeted-memory reactivation. (a) The basic comparison between cued and uncued showed reduced activity in left caudate (−20, 24, −10) for the cued sequence. (b) SWS was associated with enhanced activation in bilateral caudate (16, 8, 20 and −12, 20, 12), and bilateral hippocampi (26, −34, 2 and −22, −34, 6) for the cued sequence relative to the uncued. (b) REM sleep was associated with cueing related activity enhancement in left cerebellum (−32, −54, −44 and 20 −72, −26), left superior parietal cortex (−28, −56, 68 and 22, −54, 38), left sensorimotor cortex (SMC) (−40, −32, 68), left dorsolateral prefrontal cortex (dlPFC) (−30, 34, 28) and right premotor cortex (PMC) (42, −2, 32 and 42, −2, 58). These findings were whole brain corrected (*p* < 0.05) and displayed as sagittal and coronal projections superimposed on a standard Montreal Neurological Institute (MNI) brain. Colour bar indicates t-values. Anatomical labelling based on peak z-score location.

To examine the neural responses associated with a procedural memory that has undergone TMR during sleep, we contrasted activity during performance of the cued sequence with performance of the uncued sequence at retest (*p* < 0.05, whole brain corrected). This basic contrast revealed no increases, but a decrease in activity for the cued sequence in a cluster-spanning left caudate and anterior cingulate gyrus, and a second left occipital/cuneus cluster (Fig 3A).

Next, we investigated whether other factors were related to the cueing effect by running the same cued > uncued contrast again with five regressors added at the second level: duration (mins) of SWS (SWStime), duration of REM (REMtime), duration of stage 2 (Stage2time), the number of replayed sequences (replays) and the procedural cueing effect. Prior work has shown that the length of SWS predicts behavioural consolidation effects after both normal sleep [45] and TMR [46,47]. Our cues were presented during SWS; therefore, we expected this regressor to reveal cueing effects in regions that underscore motor learning. As hypothesised, the contrast [(cued > uncued) * SWStime] showed significantly stronger responses for the cued sequence in bilateral caudate and hippocampi (Fig 3B, Table 3 for complete list of responses). We observed significantly decreased activity for the cued sequence in bilateral sensorimotor cortex (SMC), and right frontal eye fields (FEF) (Table 4). In sum, a longer duration of SWS was associated with increased responses to the cued sequence within predicted subcortical regions that are critical to procedural learning (caudate and hippocampus), while other key cortical motor regions showed decreased responses (SMC and FEF).

**Table 3:** Coordinates of local maxima for brain regions showing greater activity for the cued relative to the uncued sequence (*n* = 20), when considering covariates of SWS, REM, stage 2, replays, and the procedural cueing effect.

Region	MNI x, y, z(mm)	No. of voxels	Peak T	Peak Z	Peak P(unc)
(Cued > Uncued) * SWS duration (mins)					
Right caudate	16, 8, 20	606	8.49	4.97	<0.001
Left caudate	−12, 20, 12	624	6.22	4.24	<0.001
Right heschl’s gyrus	44, −16, 8	77	5.06	3.75	<0.001
Right hippocampus	26, −34, 2	139	4.3	3.38	<0.001
Left hippocampus	−22, −34, 6	128	3.74	3.06	0.001
(Cued > Uncued) * REM sleep duration (mins)					
Right superior parietal lobe	22, −54, 38	677	5.98	4.15	<0.001
Left dlPFC	−30, 34, 28	342	5.03	3.74	<0.001
Left superior parietal lobe	−28, −56, 68	106	4.87	3.66	<0.001
Left fusiform gyrus	−40, −72, −10	74	4.71	3.59	<0.001
Right PMC	42, −2, 32	123	4.69	3.58	<0.001
Left cerebellum	−8, −70, −28	107	4.41	3.43	<0.001
Left somatosensory cortex	−40, −32, 68	79	4.38	3.42	<0.001
Left cerebellum	−32, −54, −44	73	4.15	3.3	<0.001
Right cerebellum	20, −72, −26	119	3.98	3.2	0.001
Right PMC	42, −2, 58	80	3.88	3.15	0.001
Left supramarginal gyrus	−58, −44, 30	83	3.74	3.06	0.001
Right middle temporal gyrus	54, −56, 10	112	3.63	3	0.001
Left superior temporal gyrus	−44, −38, 20	51	3.62	2.99	0.001
Right cerebellum	14, −78, −50	66	3.4	2.86	0.002
Right supramarginal gyrus	48, −38, 36	54	3.29	2.79	0.003
(Cued > Uncued) * stage 2 sleep duration (mins)					
Right caudate	16, 8, 22	223	6.1	4.19	<0.001
Left cingulate gyrus	−14, −14, 34	83	4.01	3.22	0.001
(Cued > Uncued) * replayed sequences					
Right primary motor cortex	62, 2, 38	84	4.71	3.59	<0.001
(Cued > Uncued) * procedural cueing effect (cued minus uncued RT)					
Right orbitofrontal cortex	16, 38, −8	51	4.73	3.6	<0.001
Bilateral midbrain	0, −18, −16	86	4.56	3.51	<0.001
Right anterior cingulate	20, 40, 14	133	4.23	3.34	<0.001
Right middle frontal gyrus	36, 10, −36	159	4.19	3.32	<0.001
Left anterior cingulate	−2, 4, −6	108	4.07	3.25	0.001

The main effect of TMR across the whole brain, showing increased activity when considering SWS duration, REM sleep duration, stage 2 sleep duration, replays and the procedural cueing effect, voxel threshold of *p* = 0.05 (whole brain corrected) and extent threshold of k > 50 voxels. All active voxels are positive for the cued > uncued comparison. There were no significant increases for the cued > uncued simple contrast.

**Table 4:** Coordinates of local maxima for brain regions showing decreased activity for the cued relative to the uncued sequence (*n* = 20), when considering covariates of SWS, REM, stage 2, and replays.

Region	MNI x, y, z(mm)	No. of voxels	Peak T	Peak Z	Peak P(unc)
(Uncued > Cued)					
Left caudate	−20, 24, −10	86	4.09	3.42	<0.001
Left occipital lobe	−26, −102, 4	111	4.07	3.41	<0.001
(Uncued > Cued) * SWS duration (mins)					
Left somatosensory cortex	−62, −16, 40	80	4.62	3.54	<0.001
Right somatosensory cortex	62, −10, 42	89	4.14	3.29	0.001
Right middle frontal gyrus	42, 28, 50	55	4.02	3.22	0.001
(Uncued > Cued) * REM sleep duration (mins)					
Right caudate	22, 24, 20	248	5.42	3.92	<0.001
(Uncued > Cued) * stage 2 sleep duration (mins)					
Right subgenual cingulate	8, 8, −14	96	4.75	3.61	<0.001
Left primary motor cortex	−38, −32, 70	51	4.1	3.27	0.001
(Uncued > Cued) * replayed sequences					
Right caudate	14, 8, 18	69	5.34	3.88	<0.001
Right middle temporal gyrus	50, −34, −8	253	4.47	3.46	<0.001
Right precuneus	20, −40, 0	84	3.54	2.94	0.002

The main effect of TMR across the whole brain, showing decreased activity that was associated with SWS duration, REM sleep duration, stage 2 sleep duration, replays, and the procedural cueing effect, voxel threshold of *p* = 0.05 (whole brain corrected) and extent threshold of k > 50 voxels. All active voxels are positive for the uncued > cued comparison. There were no significant decreases associated with the procedural cueing effect.

When time spent in stage 2 was examined [(cued > uncued) * Stage2time] the pattern of activity was similar to that observed with the SWS regressor, with significantly increased activity for the cued sequence in the same right caudate cluster, and decreased activity in left SMC.

REM has also been linked to motor sequence memory reactivation and consolidation [24,25,37–39,42]; therefore, we next considered TMR effects with REMtime as the regressor of interest [(cued > uncued) * REMtime]. This showed significant clusters of increased activation for the cued sequence in a number of motor regions, including bilateral cerebellum, right premotor cortex (PMC), and left SMC (Fig 3C, Table 3). Additionally, left dorsolateral prefrontal cortex (dlPFC) and bilateral superior parietal clusters also showed greater activity for the cued sequence. Interestingly, this regressor also revealed decreased activity for the cued sequence in a right caudate cluster, which overlapped with the right caudate cluster that showed increased activity when considering SWS (Table 4). Thus, REM and SWS were associated with changes in activity after cueing in separable motor learning regions and also had opposite relationships with activity levels in the right caudate.

The number of sequences that were played as cues during sleep could potentially have influenced changes in functional activity. When examining the number of replays as the second-level regressor [(cued > uncued) * replays], we found increased activity in the right SMC, indicating that more replays were associated with a larger response for the cued sequence in these areas. Conversely, activity in the right caudate decreased for the cued sequence; therefore, the replays regressor showed the reverse pattern to the SWS regressor.

Next, the amount of overnight improvement in performance has previously been linked to neurophysiological changes [7,8,10–12,35,50]; therefore, we also examined the procedural cueing effect as a regressor [(cued > uncued) * procedural cueing effect]. This did not identify increases in any of the predicted regions (Table 3), and there were no significant decreases.

### Functional Connectivity Analysis

After identifying localised activation differences associated with TMR during SWS, we sought to examine the functional connectivity of task-related regions that showed sensitivity to TMR. This was achieved with four psychophysiological interaction (PPI) analyses seeded in right and left hippocampus and right and left caudate nucleus, all based on peak coordinates identified in relation to the SWStime regressor [(cued > uncued) * SWStime]. Each analysis explored how connectivity from the seed region to the whole brain differed between cued and uncued sequences (*p* < 0.05, whole brain corrected).

**Fig 4:**
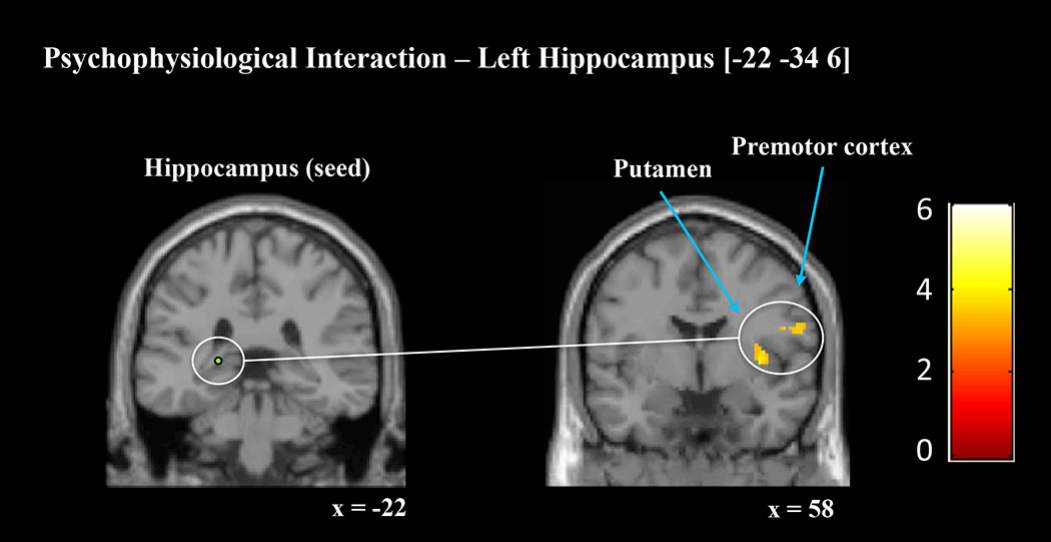
Regions of increased functional connectivity after TMR. A PPI analysis revealed enhanced connectivity for the cued sequence between left hippocampus (−22, −34, 6) and right putamen (36, −2, 4) and PMC (58, 4, 22). Contrasts displayed as sagittal and coronal projections superimposed on a standard MNI brain. Colour bar indicates t-values. Anatomical labelling based on peak z-score location.

Crucially, both hippocampal seeds showed enhanced connectivity with key motor regions during cued relative to uncued sequence performance. Left hippocampus (−22, −34, 6) showed greater connectivity to right PMC, right putamen, left putamen, and thalamus, and a cluster spanning bilateral thalamus and midbrain (Fig 4, Table 5). Connectivity between this seed and bilateral middle temporal gyrus was also enhanced. The right hippocampus seed (26, −34, 2) showed greater connectivity with left caudate and right fusiform face area (FFA). The left caudate seed (−12, 20, 12) showed enhanced connectivity with bilateral thalamus, right temporal pole, left FFA, and left lingual gyrus. The right caudate seed showed increased connectivity with left superior parietal cortex. There was just one instance of reduced functional connectivity for the cued sequence, between the left caudate seed (−12, 20, 12) and left cerebellum. To summarise, TMR was associated with increased subsequent connectivity and activation within regions associated with procedural learning. This altered pattern of brain activity may underpin the behavioural enhancements we observed.

**Table 5:** Coordinates of local maxima for brain regions showing increased (cued > uncued) and decreased (uncued > cued) functional connectivity (PPI) after TMR (*n* = 20).

Region	MNI x, y, z(mm)	No. of voxels	Peak T	Peak Z	Peak P(unc)
Cued > Uncued					
					
*Seed*: *Left caudate nucleus*	−12, 20, 12				
Bilateral thalamus and midbrain	6, −22, −4	1,108	6.76	4.77	<0.001
Right temporal pole (middle)	34, 6, −38	124	5.41	4.16	<0.001
Left fusiform gyrus	−46, −54, −20	75	4.15	3.46	<0.001
Left lingual gyrus	−32, −92, −14	57	4	3.36	<0.001
					
*Seed*: *Right caudate nucleus*	16, 8, 20				
Left superior parietal cortex	−14, −76, 60	127	5.13	4.01	<0.001
					
*Seed*: *Left hippocampus*	−22, −34, 6				
Left middle temporal gyrus	−46, −32, −6	168	4.53	3.68	<0.001
Right putamen and insula	36, −2, 4	102	3.82	3.25	0.001
Left putamen and thalamus	−26, −24, 0	55	3.76	3.21	0.001
Right PMC	58, 4, 22	62	3.72	3.19	0.001
Right middle temporal gyrus	56, −36, −10	71	3.52	3.05	0.001
Bilateral midbrain and thalamus	2, −16, −2	78	3.52	3.05	0.001
					
*Seed*: *Right hippocampus*	26, −34, 2				
Right fusiform gyrus	34, −56, −16	85	4.48	3.66	<0.001
Left caudate	−4, 26, 2	53	4.18	3.47	<0.001
					
Uncued > Cued					
**					
*Seed*: *Left caudate nucleus*	−12, 20, 12				
Left cerebellum	−24, −76, −36	60	4.96	3.93	<0.001
					

PPI analysis of connectivity between four seed regions and the rest of the brain, voxel threshold of *p* = 0.05 (whole brain corrected) and extent threshold of k > 50 voxels.

## Discussion

We show that targeted reactivation of a procedural memory alters functional activity and connectivity of motor memory networks in the human brain. The enhanced response speed for the cued sequence occurred alongside increased caudate and hippocampal responses associated with time spent in SWS, and increased hippocampal-caudate connectivity. Furthermore, REM sleep was linked with cueing-related activity changes in additional motor regions including SMC, PMC, and cerebellum. Together, these results support distinct contributions of different sleep stages to consolidation, with SWS facilitating consolidation in key subcortical regions (striatum and hippocampus) that are known to support sequence learning [2–4,7–13,35], while REM sleep facilitates consolidation in cortical and cerebellar networks that are specific to motor learning (cerebellum and motor cortical regions) [2–5,7–9].

The finding that REM is associated with changes in functional activation, even though TMR occurred during preceding periods of SWS, is of particular significance because it suggests a link between NREM memory reactivation and the processing that occurs in subsequent REM sleep. REM’s role in the reactivation and consolidation of procedural memories is controversial [36,41]. It was initially linked to procedural learning by some experimental findings [37–39], but most examples of neuronal replay occur in NREM sleep [18–21,23,42], and the targeted reactivation of procedural memories has only succeeded with cues presented during NREM [28,30,31]. As a result, prominent models of reactivation and systems consolidation prioritise NREM processes [14,15,17]. The idea of interacting periods of SWS and REM was first put forward by the “sequential hypothesis” [43], which proposes that REM strengthens and stabilises processes begun during preceding SWS periods, enabling synaptic consolidation to stabilise memories after being reorganised during SWS [14]. Our findings support this model and indirectly suggest that reactivated procedural memories may undergo processing in separable REM and SWS networks.

Importantly, we found cueing-related increases in the responses of bilateral caudate nuclei and hippocampi that were associated with time in SWS. Long-term systems consolidation for motor skills is characterised by a gradual shift of the representation from caudal to ventral striatum, alongside decreasing recruitment of corticocerebellar circuits [4]. However, procedural memory consolidation over just one night tends to follow a pattern of changes very similar to that of our cued sequence, involving increased responses in corticostriatal networks [7–11,35]. The increase in hippocampal response for the cued sequence is important for several reasons. Such enhanced activity is associated with overnight changes in motor sequence performance [7,10,35], and the hippocampus is hypothesised to be critical for engaging sleep-dependent sequence consolidation [53]. Such plasticity within striatum and hippocampus could potentially be driven by spontaneous reactivation during sleep [18,20,24,25], supported by our observation that TMR during sleep can bias functional changes in these regions.

Interactions between the striatum and hippocampus have been suggested to underscore procedural learning and consolidation, perhaps mediated by connections with mPFC [12,35]. Consistent with this, we found that TMR during sleep influenced striato-hippocampal connectivity, with enhanced connectivity between right hippocampus and left caudate, and between left hippocampus and a cluster-spanning bilateral thalamus and putamen. This supports the idea that TMR during SWS can modify connectivity patterns within the brain networks underpinning performance [33], and extends this to procedural memory. This result was predicted based on prior MSL research by Albouy and coworkers [10,11,35], showing that interactions between striatum and hippocampus are associated with overnight performance gains. The mPFC is thought to mediate between hippocampus and striatum during skill acquisition and consolidation [12,35]. Neither activity nor connectivity in mPFC differed significantly between our sequences at retest, but this region may still have played a role during acquisition and consolidation. Together, these findings suggest that the functional interaction between hippocampus and striatum underscores consolidation of MSL, and our data support the idea that reactivation during SWS is the mechanism for this process. We propose that the changes in striatohippocampal connectivity reflect stabilisation and perhaps reorganisation of the sequence memory representation, which allows faster motor output when performing the sequence at retest.

Our observation that TMR-dependent changes in caudate and hippocampal activity were related to SWS adds to our previous finding that SWS is linked to the impact of TMR on behavioural measures [46], and that a shorter period of SWS containing TMR provides the same offline gains as a longer period of SWS [47]. We now additionally suggest that the amount of SWS obtained also determines the extent of neural reorganisation in relation to reactivation of a procedural memory.

While the SWS-associated functional increases for the cued memory occurred in subcortical regions known for their roles in many types of learning, REM-associated increases related to cortical and cerebellar regions that are for the most part motor learning specific. These include increased activity in bilateral cerebellum, right ventral PMC and left SMC. SMC is critical for processing complex finger movements [55], while PMC forms a loop with the striatum and thalamus during motor performance and is thought to interact dynamically during learning to create a sequence memory [4]. Our findings are consistent with other demonstrations of increased cerebellar activity after sleep compared to wake [7], which may indicate a higher demand for error monitoring after consolidation [56]. Collectively, these regions comprise much of the corticocerebellar network, including SMC, ventral PMC, and cerebellum [57], which may suggest a preference for corticocerebellar processing during REM sleep. At this relatively early stage of systems consolidation, increased activity in these areas may facilitate faster motor output of the cued sequence. Additionally, we observed increased activity for the cued sequence in dlPFC and superior parietal cortices in association with REM. These regions are known to interact with the striatum during early MSL and consolidation [4,58,59], so this may reflect the formation of an efficient cued sequence representation.

Interestingly, REM and SWS were linked to opposing patterns of activity in SMC and striatum: cueing-related SMC responses decreased in association with SWS and increased in association with REM, while right caudate activity decreased in association with REM and increased in association with SWS. Furthermore, an additional motor cortical region involved in directing eye movements and visual attention, the FEF [60], showed decreased activation for the cued sequence in relation to SWS. These results could indicate a competitive interaction between subcortical SWS and cortical REM networks, although future research should examine this by manipulating reactivation in both sleep stages.

Our connectivity analysis also revealed that for the cued sequence, the left hippocampus was more connected to right PMC, and bilateral thalamus and midbrain. Thalamus and striatum form a segregated loop with cortical motor regions in order to process motor information [4], alongside contributions from midbrain nuclei [61]; therefore, this result may indicate enhanced hippocampal contributions to this circuit after TMR. This enhanced connectivity may be the consequence of communication between hippocampal and cortical regions during reactivation, a process that is proposed to underscore systems consolidation during sleep [14]. Additionally, the left caudate was more connected to bilateral thalamus and midbrain for the cued sequence, while the right caudate was more connected to left superior parietal cortex. This agrees with recent observations that connectivity between striatum, thalamus, and superior parietal cortex were involved in MSL acquisition and consolidation [9]. Tighter coupling of these motor circuits as a result of TMR may support enhanced performance of the cued sequence. We found only one example of reduced functional connectivity for the cued sequence, between the left caudate and left cerebellum, and this decoupling of cerebellum is often observed as motor skills become automatized [3,4]. Since this analysis utilised seed regions from the SWS regressor, it could potentially identify whether parts of the SWS network were driving the REM network regions. However, there were no overlapping clusters from the REM regressor and the PPI; therefore, the relationship between the two networks remains unclear, and this is perhaps a question future studies should explore by manipulating both sleep stages and replay. In sum, these findings demonstrate that cued reactivation influences functional interactions across a range of networks involved in MSL.

Notably, although longer SWS duration was associated with enhanced response in bilateral caudate nuclei after TMR, comparison of cued and uncued sequences without any additional covariates showed decreased activity in left caudate. An overnight decrease in striatal response is in line with some prior MSL studies that contrast the brain activity associated with sleep and wake retention intervals [6,12], although others show activation increases [7,8,10]. These disparate findings most likely stem from subtly different designs. Our current design differs still more, due to its focus on sleep stages and their interaction with consolidation after TMR. However, we acknowledge that by introducing regressors such as SWStime into our analyses, we potentially confound our findings with other factors that relate to these regressors, such as prior sleep pressure.

The stage 2 regressor identified a small number of regions that were also associated with SWS, with increased activity for the cued sequence in the right caudate and decreased activity in left SMC. Stage 2 duration has previously been correlated with procedural consolidation [48,49], as have the sleep spindles that are a prominent feature of this stage [49,62,63] (S1 Text), and some have proposed that reactivation during stage 2 provides the optimal neurophysiological conditions for systems consolidation [15]. The overlap we observed between results for SWS and stage 2 suggests that similar forms of consolidation may occur in these regions across the entirety of NREM sleep. This prompted us to examine NREM sleep as a whole (SWS+S2 duration). The NREM covariate provided virtually identical results to the SWS covariate, indicating a strong influence of SWS (S1 Text).

The number of sequences played to participants was positively correlated with SWS duration (r = 0.49, *p* = 0.03) due to our opportunistic method of cueing across SWS epochs during the first half of the night (i.e., participants with more early night SWS received more cues); therefore, both were included in the fMRI model to identify the unique variance associated with each. We had no a priori hypotheses for this regressor, since the number of cues that are presented during sleep has previously shown no relationship with the behavioural or neural effects of TMR [27–33,46,47]. Unexpectedly, replays were linked with a small subset of regions that were also associated with REM sleep, with increased activity in right SMC and decreased right caudate when performing the cued sequence. This result suggests that the number of reactivation cues does influence the physiological consolidation process, despite not correlating with behavioural measures of consolidation. The similarity between this result and the REM regressor could even suggest that the extent of cueing in NREM determines subsequent processing of those memories in REM sleep.

Neuronal firing sequences in rodents [18,20–23,42] and regional blood flow changes in humans [24–26] have both demonstrated spontaneous learning-related reactivation during sleep in areas that are critical to learning, including the hippocampus [18,26,42], striatum [20,24,25], and motor cortical regions [21,23]. Recently, reactivation in rodent primary motor cortex was linked to acquisition and sleep-dependent consolidation of a new motor skill, as well as subsequent changes in neuronal firing properties [21]. Coupled with this, TMR has been shown to bias learning-specific neuronal firing sequences during sleep in rodents [19] and trigger similar neural responses in the hippocampus and parahippocampus during SWS in humans [27,33]. Reactivation in the current study very likely engages similar processes, and this activity during NREM paves the way for the altered brain function observed after sleep. Together, these findings convincingly couple learning-related reactivation during NREM with memory consolidation. However, the mechanism by which REM sleep then acts upon a reactivated memory trace remains to be explored.

There are some limitations to the current work. Our behavioral cueing effect was apparent during early blocks at retest but not later blocks where our sequence-specific measure was calculated. As a result, we cannot be certain whether the cueing effect at early sequence blocks was due to unspecific improvements in sensorimotor mapping or sequence learning. Thus, it is possible that TMR integrated the tones more tightly with the cued sequence, allowing faster responses. Prior work in which only sequence-specific skill has been shown to benefit from sleep-dependent consolidation [51–53] and TMR during sleep [28,31] provides some confidence that the difference we observed was sequence specific. The lack of an effect at later blocks most likely reflects the cued sequence approaching ceiling performance prior to the uncued sequence.

It should also be noted that the two sequences share some basic features (e.g., triplets such as 2-4-3), meaning it is possible that TMR benefitted these features in the uncued sequence. The fact that we find significant performance differences in early blocks and in our prior work [28] suggests the sequences did form separate memory representations that could be cued separately, despite these shared features. A procedural task with more separable memory representations might produce more robust cueing effects at the neural and behavioral level.

To conclude, we show that TMR of a procedural memory alters functional activity and connectivity changes in striatum and hippocampus, and this altered function may explain the behavioural effects associated with TMR. We provide tantalising hints that REM sleep, as well as SWS, after TMR is important for neural changes that support enhanced postsleep performance of a procedural skill. These findings further our understanding of sleep’s unique role in memory consolidation by showing that offline skill enhancement depends on the reactivation of specific memory traces, and the associated changes in neural activity rely upon processing that may unfold across several subsequent stages of sleep.

## Materials & Methods

### Participants

Twenty-five (16 males) healthy participants aged 18–35 y (mean age = 23.8 y, SD ± 4.2) volunteered. Three were excluded because of ceiling performance at learning, falling asleep during the fMRI scanning session, and disrupted SWS as a result of cueing. Data from the remaining twenty-two (14 male) participants aged 18–35 y (mean age = 23.5 y, SD ± 4.3) were analysed. Prestudy questionnaires determined that participants had no history of psychiatric diseases, neurological, sleep, or motor disorders and kept a normal sleeping pattern in the week prior to the experiment. Participants were free of any form of medication, except for females using the contraceptive pill. They were asked to abstain from caffeine and alcohol 24 h prior to testing and between test sessions and to avoid napping on the experimental day. All participants were right-handed, confirmed by a score of 80% or more on the Edinburgh Handedness scale [64]. Informed written consent was acquired in accordance with the University of Manchester (ID: 11367) and the University of Liverpool (ID: RETH000585IREC) ethics committees. Prior to the scanning session, a qualified radiographer from the University of Liverpool screened participants to assess their suitability for MRI.

### Experimental Task and Design

Participants arrived at 7–8pm for the first session and were fitted for polysomnography (PSG). They then performed an adapted SRTT [44] containing psuedorandomly interleaved blocks of two 12-item sequences, A (1-2-1-4-2-3-4-1-3-2-4-3) and B (2-4-3-2-3-1-4-2-3-1-4-1), with no runs of more than two blocks of the same sequence (Fig 1). Sequences followed the constraints that each item appeared three times, was present once in each half of the sequence, and could not appear sequentially (e.g., 1–1). Blocks containing three repetitions of the sequence were separated by a consistent 15-s gap where RT and error rate feedback was presented. Sequences A and B were counterbalanced across cueing conditions so that half were cued during sleep with sequence A and half with sequence B.

Each sequence was accompanied by pure tones, four high-pitched tones were used for one sequence (fifth octave; A/B/C#/D), and four lower pitched tones were used for the other (fourth octave; C/D/E/F). Participants performed 20 blocks of each sequence, followed by four random blocks containing no repeating sequence, (“R” displayed centrally), containing high- (two blocks) and low-pitched tones (two blocks)

Trials contained an auditory tone and visual cue in one of four spatial locations, corresponding to a four-button box used with all fingers of the left hand (Fig 1). “A” or “B” appeared centrally on the screen to indicate the sequence. Participants were asked to respond as quickly and accurately as possible and were not asked to explicitly learn the sequences. Visual cues were objects or faces appearing in the same position for both sequences. Participants were told the nature of cues (objects/faces) was irrelevant. Stimuli remained on screen until a correct response was made, followed by a 300 ms intertrial interval.

Participants were invited to sleep overnight in the Neuroscience and Psychology of Sleep (NaPS) Laboratory at the University of Manchester, where they were monitored with PSG. Lights out was at 11pm. Brown noise was presented throughout the night. During periods of SWS, one sequence’s tones were replayed just loud enough to be audible above the brown noise (at approximately 48 dB), in the same order as learning and at a speed similar to mean presleep performance, in blocks of 2 min replay (CUE), followed by 2 min silence (NO-CUE). Replay was only instigated after 3 min of what the experimenter considered to be stable SWS, in line with AASM criteria. Sequence A and B were counterbalanced across cued and uncued conditions, and tones (high/low pitch) were counterbalanced across sequences. Cues were stopped upon signs in the EEG of arousal or leaving SWS.

Participants were woken up at 7–8am. The retest session took place 11am–12pm during fMRI, consisting of 24 sequence blocks (12 cued and 12 uncued), followed by 24 random blocks (12 blocks containing cued tones and 12 containing uncued tones). “REST” was displayed centrally during 15 s breaks between blocks. Order of learning (i.e., whether participants began a session with sequence A or B), replay, and retest was counterbalanced across participants. Lastly, free recall was measured outside the scanner with participants marking sequence order on paper. The Stanford Sleepiness Scale assessed alertness prior to learning and retest sessions [65].

### Equipment

All experimental scripts were executed using MATLAB 6.5 (The MathWorks Inc., Natick, MA, 2000) and Cogent 2000 (Functional Imaging Laboratory, Institute for Cognitive Neuroscience, University College, London). Sounds were presented via a pair of Sony noise cancelling headphones during the learning session, via PC speakers during sleep replay, and via an MR compatible headphone system (MR Confon) during retest (fMRI). A serial four-button box attached to a Domino multicontroller from Micromint recorded participant responses, with a time resolution of approximately 1 ms.

### fMRI Data Acquisition

Functional MRI data were acquired using an eight-channel head coil with a Siemens 3T Allegra MR scanner. The BOLD signal was recorded with T2*-weighted fMRI images obtained via a gradient echo-planar imaging (EPI) sequence. We acquired 50 oblique transaxial slices at 25-degree tilt, in an ascending sequence, voxel size 3 x 3 x 2.8 mm, matrix size of 64 x 64, flip angle of 80 degrees, repetition time (TR) of 2,960 ms, and echo time (TE) of 30 ms. A structural T1-weighted image was also acquired, using a 3D IR/GR sequence with a matrix size of 224 x 256 x 176, cubic voxels with isotropic resolution of 1 mm^3^, TR of 2,040 ms, TE of 5.57 ms, inversion time of 1,100 ms, and flip angle of eight degrees.

### Behavioural Analysis

Three measures of behavioural performance improvement were calculated by comparing presleep to postsleep performance in terms of both accuracy and RT: (1) Early sequence improvement: mean performance on the first four blocks of SEQ_C and SEQ_U at retest subtracted from mean performance on the last four blocks of SEQ_C and SEQ_U at learning. This measure identifies whether any cueing effects are present immediately upon retest. (2) Late sequence improvement: mean performance on the last four blocks of SEQ_C and SEQ_U at retest subtracted from mean performance on the last four blocks of SEQ_C and SEQ_U at learning. This measure identifies whether any cueing effects are present toward the end of the retest session. (3) Sequence-specific improvement: this utilised a well-established method to separate sequence skill from learning of sensorimotor mapping between response keys and visual stimuli, by subtracting sequence from random performance [28,52–54]. For the learning session, we subtracted performance on the last four blocks of SEQ_C and SEQ_U from the two blocks of RAND_C and RAND_U that also occurred at the end of the learning session. For the retest session, we subtracted the last four blocks of SEQ_C and SEQ_U from the first four blocks of RAND_C and RAND_U, which were performed immediately afterwards. Lastly, we calculated “sequence-specific improvement” by subtracting the retest score from the learning score [28]. Together, these measures index the way TMR influences performance of sequences across the retest period. RTs >1,000 ms were excluded, while trials with multiple button presses prior to the correct press were included. Explicit recall was assessed in line with previous work [28], whereby participants consciously recalled sequence order and marked it on paper. Individual items within a segment containing >2 consecutive correct items and in the correct sequence position were considered correct.

We also calculated how strong the cueing effect was for each participant, in terms of their RT performance and their explicit sequence knowledge. The “procedural cueing effect” was obtained by subtracting early sequence improvement (defined above) for the cued from the uncued sequence. The “explicit cueing effect” was calculated by subtracting explicit sequence knowledge for the cued from the uncued sequence (note that this was only measured after consolidation) [28]. These behavioural measures were used to correlate with EEG and fMRI analyses.

Mixed ANOVA and paired sample *t* tests were used for planned comparisons of cued and uncued sequence RT and recall. Associations between behavioural measures and EEG features were tested with Pearson’s correlations. Where Shapiro-Wilk tests indicated a non-normal distribution, Wilcoxon signed-rank tests or Spearman’s Rho correlations were used. All statistical tests were two-tailed, significance level *p* < 0.05. All means presented in the text ± standard deviation.

### EEG Recording and Analysis

Electrodes were affixed at standard locations, F3, F4, C3, C4, C5, C6, CP3, CP4, CP5, CP6, P7, P8, O1, and O2, referenced to the combined mean of left and right mastoid, according to the 10–20 system. Also attached were left and right electro-oculagram, left and upper electromyogram and forehead ground electrode. Impedance below 5Ω was verified, and the digital sampling rate was 200 Hz. Data were scored according to standard criteria [66] by two experimenters, the second of which was blind to CUE/NO-CUE periods. Mean time spent in sleep stages (Table 2) was based on *n* = 21: one participant was excluded due to loss of EOG channels in the latter part of the night (after TMR had concluded). Welch’s method was used for power spectral density analyses, with alpha power (8–12 Hz) at occipital channels averaged over separate concatenated time series for CUE and NO-CUE periods via MATLAB 2010.

### fMRI Analysis

Functional imaging data were analysed using Statistical Parametric Mapping 8 software (SPM8; Wellcome Department of Cognitive Neurology, London, UK). The first two volumes of each functional EPI run were removed to allow for T1 equilibration. Two participants were excluded from analysis for excessive movement >3.5 mm. Functional images were realigned to correct for motion artifacts using iterative rigid body realignment, minimizing the residual sum of squares between all scans and the first scan. Functional images were then spatially normalised to the Montreal Neurological Institute brain (MNI space), resampled to voxel size 2 x 2 x 2 mm. Lastly, a spherical Gaussian smoothing kernel (full-width half maximum = 8 mm) was applied to each participant’s normalised data.

Statistical analysis of MRI data at the single subject level used the general linear model (GLM) [67]. Blocks of cued and uncued sequences were modelled as boxcar functions, and button presses for individual trials were also modelled as single events with zero duration. These were temporally convolved with the hemodynamic response function (HRF). The design matrix also included six nonconvolved head motion regressors and, lastly, baseline activation was modelled with a constant regressor. A first-order autoregressive model with added white noise was used to model serial correlations, estimated with a restricted maximum likelihood algorithm. A high pass filter was utilised by using a cut off period of 128 s, removing low frequency noise.

Contrast parameter images were generated for each participant with balanced linear t-contrasts, including one-sample *t* tests for the cued > uncued contrast. These contrast images were subsequently entered into a second-level random effects analysis. To determine whether the difference between cued and uncued sequences was associated with other factors, we included sleep parameters (minutes spent in stage 2, SWS, and REM sleep), the number of replayed sequences and the procedural cueing effect as covariates in a single second-level analysis.

All analyses were whole brain corrected via a Monte Carlo simulation [68]. This modelled the entire imaging volume across 1,000 iterations, assuming a type I error of *p* < 0.05 at a voxel-wise uncorrected threshold of *p* < 0.005, and recommended a cluster extent threshold of 51 contiguous voxels. Clusters falling entirely in white matter were not reported.

### Functional Connectivity

We examined the functional connectivity between regions using PPI. Four separate PPI’s were conducted. Each spherical seed region (radius 6 mm) was based on peak coordinates of the group response to the cued > uncued contrast with SWStime as a second-level covariate. Coordinates were chosen from the results when SWStime was the regressor of interest because this identified regions that were hypothesised a priori to show changes in functional connectivity after TMR, based on previous work [8,9,11,12,33]. For each participant, the time course of activity for a sphere with a radius of 6 mm around the peak coordinate of the seed region was extracted and deconvolved, forming the physiological factor. We were interested in how connectivity with each seed varied after TMR during sleep, therefore our psychological factor was the contrast (cued > uncued). For each participant, our PPI design matrix included three regressors: the physiological factor, the psychological factor, and the interaction (physiological versus psychological), in addition to the button press regressor convolved with the HRF, and the six nonconvolved motion regressors. Contrast images for the PPI regressor were then generated using a one-sample *t* test. These images formed a second-level random effects analysis. The results represented regions whose functional connectivity was sensitive to whether the sequence had been cued during sleep or not. PPI data was thresholded in the same manner as localised data, i.e., 51 contiguous voxels of *p* < 0.005 were considered significant at *p* < 0.05 based on our Monte-Carlo simulation. The coordinates used for the PPI analyses are listed below: Left caudate nucleus −12, 20, 12; right caudate nucleus 16, 8, 20; left hippocampus −22, −34, 6; right hippocampus 26, −34, 2.

## Supporting Information

S1 Data Summary of data. (XLSX)

S1 Text Supplementary information. SRTT performance at training, testing of explicit sequence knowledge, and correlations between behaviour and EEG features (fast spindles and slow oscillations).
(DOCX)

## Abbreviations

dlPFC: dorsolateral prefrontal cortex
EEG: electroencephalography
EPI: echo-planar imaging
FEF: frontal eye fields
FFA: fusiform face area
fMRI: functional magnetic resonance imaging
GLM: general linear model
HRF: hemodynamic response function
MNI: Montreal Neurological Institute
mPFC: medial prefrontal cortex
mPFC-HPC: medial prefrontal-hippocampal
MSL: motor sequence learning
NaPS: Neuroscience and psychology of sleep
NREM: nonrapid eye movement
PMC: premotor cortex
PPI: psychophysiological interaction
PSG: polysomnography
REM: rapid eye movement
RT: reaction time
SEM: standard error of the mean
SMC: sensorimotor cortex
SRTT: serial reaction time task
SWS: slow-wave sleep
TE: echo time
TMR: targeted memory reactivation
TR: repetition time
