# Supplementary material for: Cued Reactivation of Motor Learning during Sleep Leads to Overnight Changes in Functional Brain Activity and Connectivity
Source: PLoS Biol. 2016 May 3;14(5):e1002451. doi: 10.1371/journal.pbio.1002451 (PMC4854410; doi:10.1371/journal.pbio.1002451)
Supplement: S1 Text — SRTT performance at training, testing of explicit sequence knowledge, and correlations between behaviour and EEG features (fast spindles and slow oscillations). (DOCX) [file pbio.1002451.s002.docx]

**Cued Reactivation of Motor Learning During Sleep Leads to Overnight Changes in Functional Brain Activity and Connectivity.**

James N. Cousins, Wael El-Deredy, Laura M. Parkes, Nora Hennies, Penelope A. Lewis

**Supporting Information**

**Explicit sequence knowledge**

Participants were tested for their explicit knowledge of the two sequences in a final test outside the scanner, where they were asked to mark sequence order on paper. Shapiro-Wilk tests indicated a non-normal distribution, therefore a related-samples Wilcoxon signed-rank test was used, which showed no significant difference between cued and uncued sequence recall (p=0.68) (Supplementary Fig 1b).


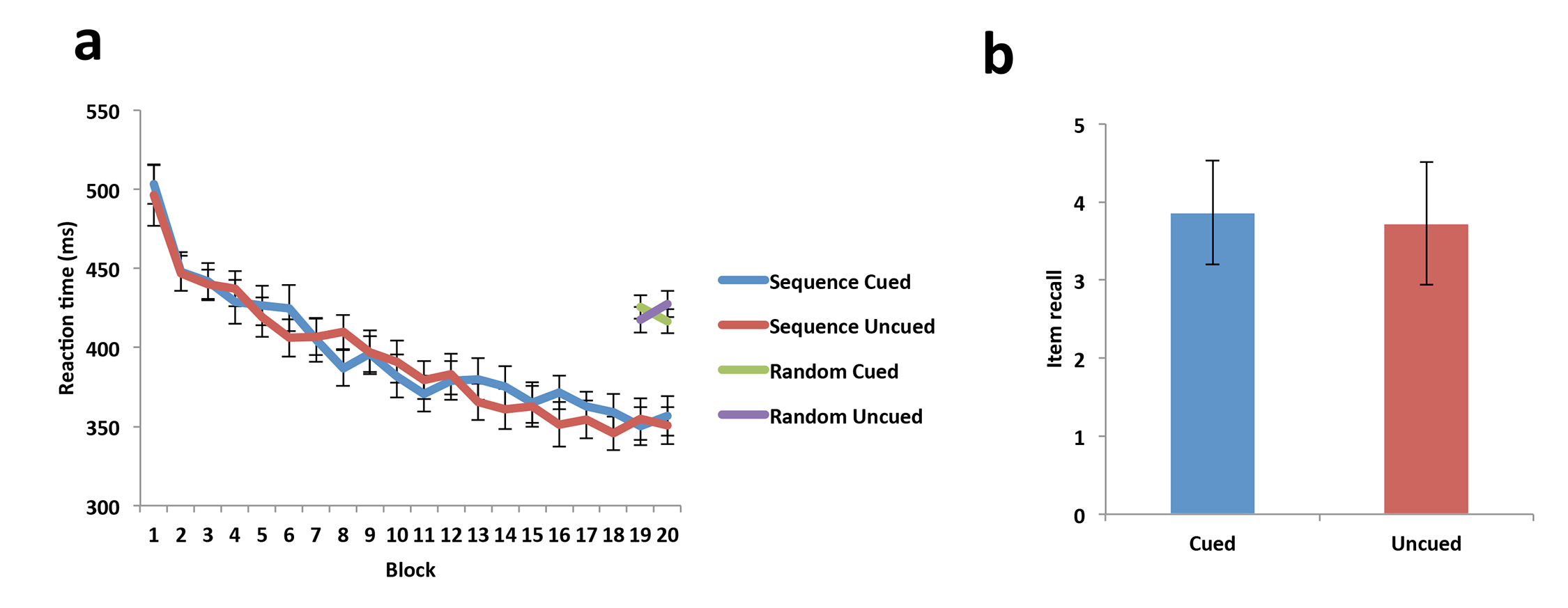


**Supplementary Fig 1: Behavioral results for SRTT training and explicit test.**(a) Prior to sleep, performance of the to-be cued and uncued sequences improved at similar rates across training. (b) Post sleep measurement of explicit sequence knowledge showed no difference in item recall between the two sequences. Error bars represent standard error of the mean (SEM).

It is unclear why this failed to replicate the findings of our previous study [1], but we tentatively suggest that some subtle methodological differences between the two studies may account for these divergent findings. Firstly, the Cousins et al. [1] learning procedure involved two sequences being performed back to back, with only a 2 sec fixation between them, followed by a 30 sec break. This was altered in the current study in order to optimize the fMRI component of the experiment. Instead there was a 15 sec gap after every sequence, during learning and retest. With this procedure, each sequence can be considered as more discreet, with a more distinct beginning and end. Moreover, participants had more time to reflect on each sequence separately during breaks. This admittedly subtle difference may have led to slightly different strategies during learning that may have influenced consolidation. Second, the context of learning and retest differed in the two studies. For Cousins et al. [1] all learning and testing occurred in the same room at the same computer, while in the current study all aspects of the retest were different, with the procedural retest taking place in the MRI scanner, and the explicit test taking place in a quiet room nearby. We did not expect these minor procedural changes to impact explicit recall, therefore further work is needed to establish under which conditions explicit knowledge emerges after procedural learning.

**EEG analysis**

We sought to characterize the relationship between the effects of TMR and key features of sleep that are linked to memory processing by examining sleep spindles and the slow oscillation. Sleep spindles are rapid bursts of relatively high frequency activity (12-15Hz) occurring in NREM sleep. The thalamic gating of external sensory processing during spindles [2] is suggested to facilitate internal memory consolidation processes [3] and spindles have been consistently linked with memory consolidation [4]. Spindles are further delineated into slow spindles (12-13.5Hz) that are linked to activity in superior frontal gyrus, and fast spindles (13.5-15Hz) that are associated with sensorimotor regions, medial frontal cortex and hippocampus [5]. Our prior study found correlations between regionally specific fast spindles and the effects of TMR on reaction times [1], therefore we performed similar analyses on the current data set.

Slow-wave sleep is defined by very low frequency oscillations (<1Hz) and delta activity (1-4Hz) [6]. The cortically generated slow oscillation comprises waves that propagate across the cortex predominantly from prefrontal regions [7]. This activity is proposed to maintain homeostasis in memory networks through synaptic downscaling [8], as well as synchronize corticohippocampal communication [9] in order to transfer long-term declarative memories from hippocampus to neocortex [10]. We previously identified relationships between slow oscillations and the effects of TMR on explicit sequence recall [1], therefore we explored these same relationships in the current study.

Electrodes were grouped for analysis into 2 ‘Frontal’ (F3 and F4), 2 ‘Parietal‘(P7 and P8) and 8 ‘Central’ (C3, C4, C5, C6, CP3, CP4, CP5 and CP6). The mean of these groupings was calculated for our EEG measures, consistent with previous work [1]. Loss of one electrode due to noise resulted in exclusion of that group from further analyses. Epochs of CUE and NO-CUE periods (2mins) were extracted for every channel, and adjacent CUE and NO-CUE periods were rejected if either contained visually identified artefacts such as movement. Epochs were then concatenated for each participant, creating a separate time series for CUE and NO-CUE periods. Welch’s method was utilized for power spectral density analyses, with power averaged over each time series for CUE/NO-CUE in EEGLAB [11] via MATLAB 2010. Frequency bands of interest were slow oscillation (0.3-1Hz) and fast spindles (13.5-15Hz). Mean slow oscillation power within the three separate electrode groups during CUE and NO-CUE periods was correlated with behavioral measures. An automated spindle detection algorithm was also used to determine fast spindle density at each electrode [12]. Previous research has identified localized spindle increases in the hemisphere that predominantly encoded the task, such as right motor regions for a left handed motor task [13], and also localized increases during TMR [14]. To explore these regional spindle effects, fast spindle power in left (non-learning) hemisphere electrodes was subtracted from right (learning) hemisphere electrodes, providing a ‘fast spindle power laterality’ measure for the three electrode groups in CUE and NO-CUE periods. The same comparison was performed with fast spindle density, providing a ‘fast spindle density laterality’ measure. These were then correlated with behavioral measures.

**Procedural cueing effect:** To establish the link between sleep EEG features and the advantage for the cued sequence at early blocks of retest, we calculated a “procedural cueing effect” for each participant by subtracting the behavioral measure of immediate sequence improvement for the cued from the uncued sequence. This provides a behavioral index of how strong the cueing effect was in each participant. We then correlated this behavioral measure with fast spindle power laterality during CUE and NO-CUE periods. Based on previous findings [1,13] we expected positive correlations over central motor regions, and indeed the procedural cueing effect was significantly predicted by fast spindle power laterality at central electrodes during both CUE (r=0.50, p=0.03) (Supplementary Fig 2a) and NO-CUE (r=0.55, p=0.02) periods. The same correlation was marginally significant with frontal electrodes during NO-CUE (r=0.44, p=0.05), but not CUE (r=0.24, p=0.31). Parietal correlations were not significant for either CUE (r=0.15, p=0.54) or NO-CUE (r=0.27, p=0.26). Correction for multiple comparisons was performed using false discovery rate correction [15] whereby each spindle measure was considered to take place with 6 other comparisons, based on 3 locations within the two time periods of interest. No correlations survived correction. The fast spindle density laterality measure did not reveal any significant correlations with the procedural cueing effect (P>0.05).


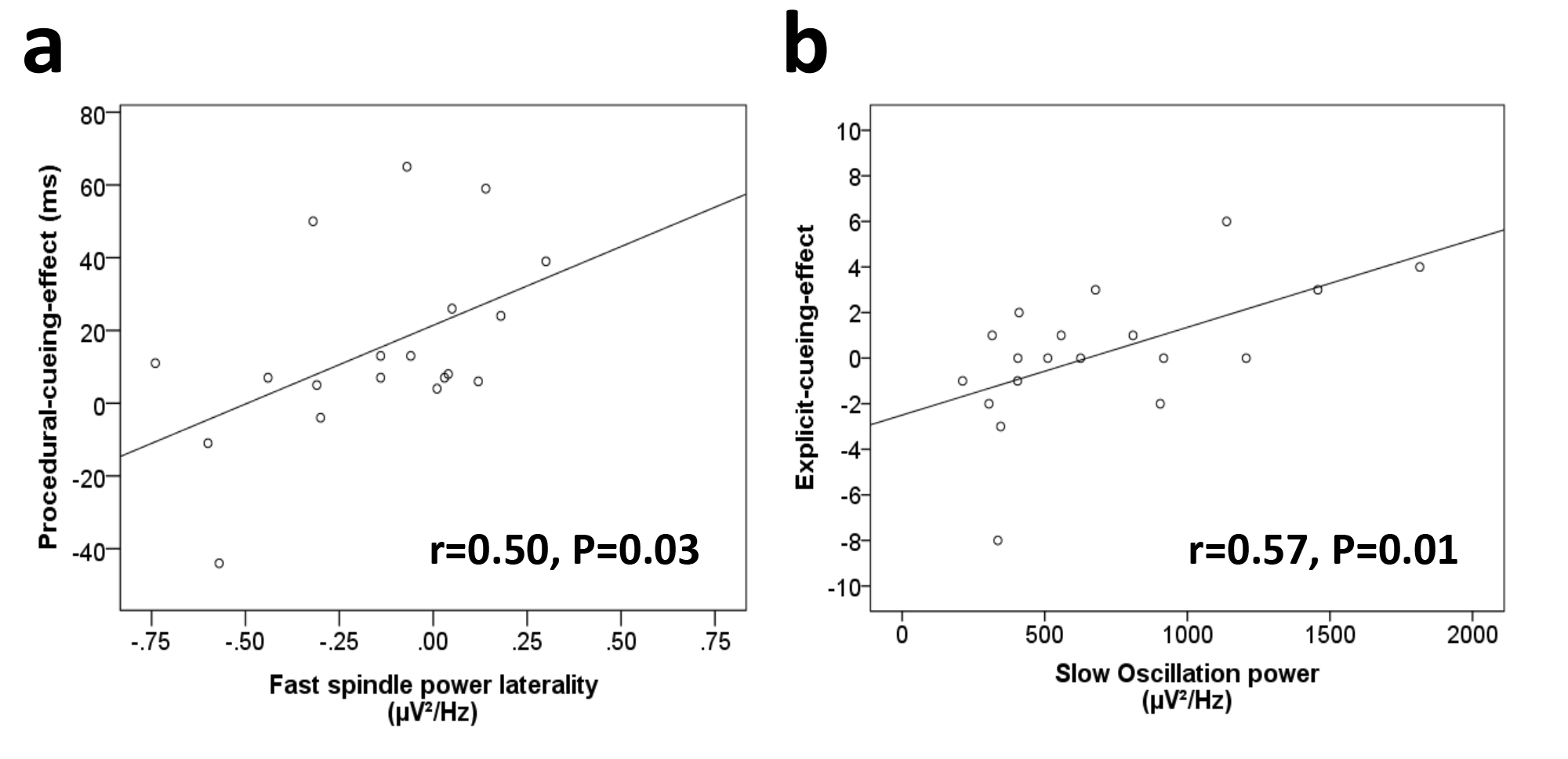


**Supplementary Figure 2: Correlations between EEG features and behavior**.
(a) Correlation between the procedural cueing effect and fast spindle power laterality at central electrodes during the CUE period (N=19). (b) The explicit cueing effect was predicted by slow oscillation power at parietal electrodes during the NO-CUE period (N=19). Correlations are presented with some participants removed (N=3) due to EEG artefacts. Error bars represent standard error of the mean (SEM).

To summarize, participants with greater power in the frequency range of fast spindles over learning hemisphere motor regions showed greater performance improvement for the cued sequence, consistant with our prior study [1]. This is also consistent with previous studies linking spindles to memory consolidation [4,13] and the proposed role of spindles in neural plasticity [10]. Fast spindles in particular are associated with activity in hippocampal and sensorimotor regions [5] and these are regions in which we observed altered activity after TMR. Others have shown an association between spindles, corticostriatal activity and sleep-dependent motor sequence consolidation [16–18]. Adding to this, our findings suggest that activity in the frequency range of fast spindles at motor cortical regions is related to consolidation of specific procedural memories. We advise caution in this interpretation however, since these correlations do not survive correction for multiple comparisons.

**Explicit cueing effect:** In order to determine if there was a relationship between sleep features and explicit knowledge of the sequence post sleep, we calculated an “explicit cueing effect” for each participant by subtracting explicit knowledge for the cued from the uncued sequence. We then correlated this with slow oscillation power (0.3-1Hz), and consistent with our prior report [1], slow oscillation power at parietal electrodes during NO-CUE predicted the explicit cueing effect (r=0.57, p=0.01) (Supplementary Fig 2b), but did not survive fdr correction. These relationships were not apparent at either frontal or central electrode sites for CUE or NO-CUE periods (p>0.05).

Despite the lack of a significant cueing effect for explicit sequence recall, we do still replicate our prior finding that individual differences in the explicit cueing effect were associated with slow oscillation power [1], although this relationship did not survive correction for multiple comparisons. A causal role for slow oscillations impacting memory consolidation has been implicated in prior studies, where the artificial enhancement of slow oscillation power enhances subsequent recall of declarative information [19,20]. Our observations tentatively support this role for slow oscillations in memory consolidation.

**Alertness**

The Stanford Sleepiness Scale [21] indicated that participants were alert during learning (Mean = 2.9 +- 0.9) and retest (Mean = 2.7 +- 0.6) and these did not differ, t(21)=0.847, P=0.41.

**fMRI analysis**

Given the similar responses associated with S2 and SWS, we tested the combined influence of these two stages (NREM sleep) (Supplementary Table 1). When considering NREM sleep as a whole [SWStime (cued > uncued) + Stage2time (cued > uncued)], results were virtually identical to the SWS covariate alone, with increased activity for the cued sequence in bilateral caudate and hippocampus, and right Heschyl’s gyrus, alongside decreased activity for the cued sequence in bilateral SMC. The size of caudate clusters reaching significance were slightly reduced for NREM sleep as a whole, while hippocampal clusters were expanded. This finding suggests a strong contribution of SWS to cueing effects in these regions.

Next, we examined the interaction between sleep stage and cueing: [SWStime (cued > uncued) > Stage2time (cued > uncued)]. This highlighted a large cluster spanning bilateral caudate, and another cluster spanning right inferior and middle temporal gyrus. The reverse interaction [Stage2time (cued > uncued) > SWStime (cued > uncued)] did not show any significant regions.

**Supplementary Table 1:** Coordinates of local maxima for brain regions showing changes in activity for the cued relative to the uncued sequence (N=20), when considering NREM sleep as a covariate (S2 + SWS), and increased activity for the cued sequence when comparing SWS to S2**.**

| **Region** | **MNI x, y, z** | **No. of** | **Peak T** | **Peak Z** | **Peak P(unc)** |
| --- | --- | --- | --- | --- | --- |
|  | **(mm)** | **voxels** |  |  |  |
| (Cued>Uncued)NREM duration (mins) | | | | |  |
| Right caudate | 16, 8, 20 | 561 | 8.49 | 4.97 | <0.001 |
| Left caudate | -12, 20, 12 | 539 | 5.65 | 4.01 | <0.001 |
| Right heschyls gyrus | 46, -14, 8 | 90 | 5.24 | 3.84 | <0.001 |
| Right hippocampus | 26, -34, 2 | 205 | 4.65 | 3.56 | <0.001 |
| Left hippocampus | -28, -38, -6 | 201 | 3.95 | 3.18 | 0.001 |
|  |  |  |  |  |  |
| (Uncued>Cued) NREM duration (mins) | | | | | |
| Left somatosensory cortex | -62, -16, 40 | 80 | 4.99 | 3.72 | <0.001 |
| Right somatosensory cortex | 62, -10, 38 | 78 | 4.0 | 3.21 | 0.001 |
|  |  |  |  |  |  |
| SWS mins (cued-uncued) > S2 mins (cued-uncued) | | | | | |
| Right caudate | 16, 8, 20 | 863 | 5.76 | 4.06 | <0.001 |
| Right inferior temporal gyrus | 60, -46, -12 | 54 | 3.42 | 2.87 | 0.002 |
|  |  |  |  |  |  |
| The main effect of targeted memory reactivation across the whole brain, showing increased activity (cued>uncued) and decreased activity (uncued>cued) that was associated with non-rapid-eye movement sleep (slow-wave sleep and stage 2 sleep duration combined), and the interaction between slow-wave sleep and stage 2 sleep duration, voxel threshold of p=0.05 (whole brain corrected) and extent threshold of k>50 voxels. The opposite interaction showed no significant differences. | | | | | |

**References**

1. Cousins JN, El-Deredy W, Parkes LM, Hennies N, Lewis PA. Cued memory reactivation during slow-wave sleep promotes explicit knowledge of a motor sequence. J Neurosci. 2014;34(48): 15870–6.

2. Schabus M, Dang-Vu TT, Heib DPJ, Boly M, Desseilles M, Vandewalle G, et al. The fate of incoming stimuli during NREM sleep is determined by spindles and the phase of the slow oscillation. Front Neurol. 2012;APR(April): 1–11.

3. Dang-Vu TT. Neuronal oscillations in sleep: insights from functional neuroimaging. Neuromolecular Med. 2012;14(3): 154–67.

4. Fogel SM, Smith CT. The function of the sleep spindle: a physiological index of intelligence and a mechanism for sleep-dependent memory consolidation. Neurosci Biobehav Rev. 2011;35(5): 1154–65.

5. Schabus M, Dang-Vu TT, Albouy G, Balteau E, Boly M, Carrier J, et al. Hemodynamic cerebral correlates of sleep spindles during human non-rapid eye movement sleep. Proc Natl Acad Sci U S A. 2007;104(32): 13164–9.

6. Amzica F, Steriade M. Electrophysiological correlates of sleep delta waves. 1998;107: 69–83.

7. Massimini M, Huber R, Ferrarelli F, Hill S, Tononi G. The sleep slow oscillation as a traveling wave. J Neurosci. 2004;24(31): 6862–70.

8. Tononi G, Cirelli C. Sleep and the price of plasticity: From synaptic and cellular homeostasis to memory consolidation and integration. Neuron. 2014;81(1): 12–34.

9. Clemens Z, Mölle M, Eross L, Barsi P, Halász P, Born J. Temporal coupling of parahippocampal ripples, sleep spindles and slow oscillations in humans. Brain. 2007;130: 2868–78.

10. Diekelmann S, Born J. The memory function of sleep. Nat Rev Neurosci. 2010;11(2): 114–26.

11. Delorme A, Makeig S. EEGLAB: an open source toolbox for analysis of single-trial EEG dynamics including independent component analysis. J Neurosci Meth. 2004;134(1): 9-21

12. Ferrarelli F. Reduced Sleep Spindle Activity in Schizophrenia Patients. Am J Psychiatry. 2007;164(3): 483.

13. Nishida M, Walker MP. Daytime naps, motor memory consolidation and regionally specific sleep spindles. PLoS One. 2007;2(4): e341.

14. Cox R, Hofman WF, de Boer M, Talamini LM. Local sleep spindle modulations in relation to specific memory cues. Neuroimage. 2014;99: 103–10.

15. Benjamini Y, Hochberg Y. Controlling the false discovery rate: a practical and powerful approach to multiple testing. J Roy Statist Soc Ser B. 1995; 289-300

16. Barakat M, Doyon J, Debas K, Vandewalle G, Morin a, Poirier G, et al. Fast and slow spindle involvement in the consolidation of a new motor sequence. Behav Brain Res. 2011;217(1): 117–21.

17. Barakat M, Carrier J, Debas K, Lungu O, Fogel S, Vandewalle G, et al. Sleep spindles predict neural and behavioral changes in motor sequence consolidation. Hum Brain Mapp. 2013;34: 2918–28.

18. Fogel SM, Albouy G, Vien C, Popovicci R, King BR, Hoge R, et al. fMRI and sleep correlates of the age-related impairment in motor memory consolidation. Hum Brain Mapp. 2014;35: 3625–45.

19. Marshall L, Helgadóttir H, Mölle M, Born J. Boosting slow oscillations during sleep potentiates memory. Nature. 2006;444(7119): 610–3.

20. Ngo HV V, Martinetz T, Born J, Mölle M. Auditory closed-loop stimulation of the sleep slow oscillation enhances memory. Neuron. 2013;78: 545–53.

21. Hoddes E, Zarcone V, Smythe H, Phillips R, Dement WC. Quantification of sleepiness: A new approach. Psychophysiology. 1973;10(4): 431-436.
